# Real‐World Outcomes of Avelumab Maintenance Therapy in Patients With Curatively Unresectable Urothelial Carcinoma in Japan: Results From the Final Analysis of Postmarketing Surveillance

**DOI:** 10.1002/cam4.71264

**Published:** 2025-10-30

**Authors:** Eiji Kikuchi, Masayoshi Nagata, Taito Ito, Masashi Sato, Mie Ogi, Makiko Morita, Masahiro Kajita, Hiroyuki Nishiyama

**Affiliations:** ^1^ Department of Urology St. Marianna University School of Medicine Kanagawa Japan; ^2^ Department of Urology Juntendo University Graduate School of Medicine Tokyo Japan; ^3^ Medical Department Merck Biopharma Co., Ltd., Tokyo, Japan, an affiliate of Merck KGaA Darmstadt Germany; ^4^ Research and Development Merck Biopharma Co., Ltd., Tokyo, Japan, an affiliate of Merck KGaA Darmstadt Germany; ^5^ Global Development Operations Merck Biopharma Co., Ltd., Tokyo, Japan, an affiliate of Merck KGaA Darmstadt Germany; ^6^ Global Patient Safety Japan Merck Biopharma Co., Ltd., Tokyo, Japan, an affiliate of Merck KGaA Darmstadt Germany; ^7^ Department of Urology, Faculty of Medicine University of Tsukuba Ibaraki Japan

**Keywords:** avelumab, effectiveness, maintenance, postmarketing surveillance, safety, urothelial carcinoma

## Abstract

**Background:**

Avelumab maintenance therapy was approved in Japan in February 2021 for the treatment of curatively unresectable urothelial carcinoma (UC) that has not progressed after prior chemotherapy based on results from the JAVELIN Bladder 100 phase 3 trial. We report the final analysis of postmarketing surveillance (PMS) data on the safety and effectiveness of avelumab maintenance in clinical practice in Japan.

**Methods:**

This prospective, multicenter, noninterventional PMS evaluated patients with curatively unresectable UC who received ≥ 1 dose of avelumab maintenance in Japan between February 2021 and December 2021. The observation period was ≤ 52 weeks in all patients. The primary objective was to evaluate the safety of avelumab maintenance based on the occurrence of prespecified adverse drug reactions (ADRs). The secondary objective was to evaluate effectiveness, including time to treatment failure (TTF), time to next‐line treatment (TTNT), and overall survival (OS).

**Results:**

Data were collected from 453 patients at 213 sites (data cutoff: March 6, 2024). Median age was 73 years, 90.3% had metastatic disease, and prior chemotherapy regimen was gemcitabine + cisplatin in 267 (58.9%), gemcitabine + carboplatin in 163 (36.0%), and others in 23 (5.1%). Any‐grade prespecified ADRs occurred in 144 patients (31.8%), including grade ≥ 3 in 35 patients (7.7%). The most common any‐grade prespecified ADRs were infusion reaction (11.7%) and thyroid dysfunction (7.3%). Median TTF was 4.6 months (95% CI, 3.8–5.3 months). Median TTNT was 9.9 months (95% CI, 8.3 months—not estimable). OS rates at 6 and 12 months were 89.2% (95% CI, 86.0%–91.8%) and 77.9% (95% CI, 73.7%–81.5%), respectively.

**Conclusion:**

Final analysis of PMS provides the largest real‐world dataset of avelumab maintenance in Japan reported to date. Findings demonstrate the acceptable safety, tolerability, and effectiveness of avelumab maintenance, consistent with data from JAVELIN Bladder 100 and real‐world studies in other countries, and support its continued use in clinical practice.

**Trial Registration:**

University Hospital Medical Information Network Clinical Trials Registry (UMIN‐CTR): UMIN 43435

## 1 Introduction

Bladder cancer is the ninth most common type of cancer worldwide, with > 613,000 new cases and > 220,000 attributable deaths in 2022 [1]. The National Cancer Center in Japan estimated that 24,800 new cases of bladder cancer and 9900 attributable deaths occurred in Japan in 2023 [2]. Urothelial carcinoma (UC) is the predominant histological type of bladder cancer, accounting for approximately 90% of all cases [3, 4]. UC may also arise in the renal pelvis, ureter, and urethra. In the US, approximately 80% of patients with UC have a primary tumor in the bladder [5], whereas in Japan, UC tumors occur with similar frequency in the upper or lower urinary tract [6, 7, 8]. Patients with unresectable locally advanced or metastatic UC (often referred to as advanced UC [aUC]) have a poor prognosis (5‐year survival rate, 8.8%) [9]. In international treatment guidelines for patients with aUC, including the Japanese Urological Association guidelines, first‐line (1L) treatment with platinum‐based chemotherapy (i.e., regimens containing cisplatin or carboplatin) is a recommended option [10, 11, 12, 13]. While most patients (approximately 80%) have a response or disease control with 1L platinum‐based chemotherapy, progression‐free survival (PFS; median, 6.3–7.1 months) and overall survival (OS; median, 13.1–14.3 months) are limited [14, 15].

In the JAVELIN Bladder 100 phase 3 trial (NCT02603432), avelumab administered as 1L maintenance in combination with best supportive care (BSC) significantly prolonged OS (primary endpoint) and PFS vs. BSC alone in patients with aUC who were progression‐free after 1L platinum‐based chemotherapy [16]. After ≥ 2 years of follow‐up in all patients, median OS from randomization in the overall population was 23.8 months with avelumab 1L maintenance + BSC vs. 15.0 months with BSC alone (stratified hazard ratio [HR], 0.76 [95% CI, 0.63–0.915]; two‐sided *p* = 0.0036), with 2‐year OS rates of 49.8% vs. 38.4%, and median PFS was 5.5 months vs. 2.1 months, respectively (stratified HR, 0.54 [95% CI, 0.46–0.64]; two‐sided *p* < 0.0001). The long‐term safety and tolerability of avelumab 1L maintenance treatment were also demonstrated [17]. In a post hoc subgroup analysis of patients enrolled in Japan (*n* = 73), efficacy and safety findings were generally consistent with those reported in the overall population (median OS: 24.7 vs. 18.7 months, respectively; unstratified HR, 0.81 [95% CI, 0.41–1.58]; median PFS: 5.6 vs. 1.9 months, respectively; unstratified HR, 0.63 [95% CI, 0.36–1.11]) [18]. Results were similar in a separate subgroup analysis that included all patients enrolled in Asia [19]. Findings from JAVELIN Bladder 100 led to the approval of avelumab 1L maintenance in various countries worldwide, and its inclusion as a recommended treatment option for cisplatin‐eligible or ‐ineligible patients with aUC in international guidelines [10, 11, 12]. Studies in various countries have confirmed the effectiveness and safety of avelumab maintenance therapy in real‐world clinical practice [20, 21, 22, 23, 24, 25, 26, 27].

In Japan, avelumab was the first programmed cell death ligand 1 (PD‐L1) inhibitor to be approved as monotherapy for curatively unresectable metastatic Merkel cell carcinoma (September 2017), and it has also been approved in combination with axitinib for curatively unresectable renal cell carcinoma (December 2019) [28]. In February 2021, avelumab was approved in Japan as maintenance therapy for patients with curatively unresectable UC who are progression‐free after prior chemotherapy, and it is recommended in Japanese Urological Association treatment guidelines [28, 29]. Because the number of patients in the JAVELIN Bladder 100 trial enrolled in Japan was limited (avelumab + BSC arm, *n* = 36) [16, 18], postmarketing surveillance (PMS) was performed in accordance with Good Post‐marketing Study Practice (GPSP) to obtain real‐world safety and effectiveness data from patients receiving avelumab maintenance therapy after regulatory approval in routine clinical practice in Japan. Here, we report the final analysis of data obtained from PMS.

## 2 Methods

### 2.1 Study Design and Patient Population

This prospective, multicenter, noninterventional PMS was a specified drug‐use results survey that was designed to evaluate the safety and effectiveness of avelumab maintenance therapy in clinical practice in Japan. All patients with curatively unresectable UC who received at least one dose of avelumab maintenance therapy in Japan between February 24, 2021, and December 7, 2021, were included. The observation period was a maximum of 52 weeks from the first dose of avelumab. Data were collected via case report forms (CRFs) and included patient and disease characteristics, prior treatment, avelumab treatment characteristics, safety, and effectiveness. Disease stage was determined by investigators per the American Joint Committee on Cancer–International Union for Cancer Control tumor–node–metastasis system.

This PMS was registered with the University Hospital Medical Information Network Clinical Trials Registry (UMIN‐CTR: UMIN 43435) and was conducted in accordance with the regulations of the GPSP Ministerial Ordinance in Japan. Approval from ethics committees/institutional review boards and informed consent for publication from patients were obtained based on the requirements of each institution [30].

### 2.2 Endpoints

The primary objective was to evaluate the safety of avelumab maintenance therapy in clinical practice in Japan. Safety data were collected for prespecified types of adverse drug reactions (ADRs) defined by the Japanese Risk Management Plan that were deemed to be associated with avelumab (adrenal insufficiency, colitis or severe diarrhea, encephalitis or meningitis, hepatic function disorders, infusion reaction [IR], interstitial lung disease, myositis or rhabdomyolysis, nerve disorders [including Guillain‐Barré syndrome], pancreatitis, pituitary disorders, renal disorders, thyroid dysfunction, and type 1 diabetes mellitus) in addition to hematuria and urinary tract infection. The secondary objective was to evaluate the effectiveness of avelumab maintenance therapy in clinical practice, including time to treatment failure (TTF; defined as time from the start of avelumab maintenance therapy to treatment discontinuation for any reason, including disease progression, prespecified ADRs, patient preference, surgery, transfer to another hospital, death, or other reasons), time to next‐line treatment (TTNT; defined as time from the start of avelumab maintenance therapy to the start of next‐line therapy or death from any cause, whichever occurred first), and OS (defined as time from the start of avelumab maintenance therapy to death from any cause).

### 2.3 Statistical Analyses

The safety analysis population was defined as all patients who received at least one dose of avelumab treatment, excluding patients who received off‐label treatment or who did not provide consent for publication. Safety data were aggregated by ADR type, severity, and time of onset and were presented as overall frequencies and percentages. The effectiveness analysis population was defined as all patients in the safety analysis population, excluding any patients transferred to a different hospital who could not be linked to pretransfer information. Effectiveness data, including TTF, TTNT, and OS, were estimated using the Kaplan–Meier method (including medians and rates), and 95% confidence intervals (CIs) were calculated. Data for patient and disease characteristics, prior chemotherapy, and avelumab treatment characteristics (including duration of treatment) were analyzed using descriptive statistics.

## 3 Results

### 3.1 Patients and Treatment

At the database lock for the final analysis (March 6, 2024), CRFs had been collected from 465 patients at 213 institutions. After the exclusion of patients who did not provide consent for publication (*n* = 9) or received off‐label treatment (*n* = 3), 453 patients were evaluable in both the safety and effectiveness analysis populations. Median age at baseline (start of avelumab treatment) was 73 years (range, 21–91 years); 75 patients (16.6%) were aged ≤ 64 years, 198 (43.7%) were aged 65–74 years, and 180 (39.7%) were aged ≥ 75 years (Table 1). Eastern Cooperative Oncology Group (ECOG) performance status was 0 in 325 patients (71.7%), 1 in 111 (24.5%), and ≥ 2 in 17 (3.8%). Primary tumor site was bladder in 244 patients (53.9%) and upper urinary tract in 209 patients (46.1%). Comorbidities at baseline included renal impairment in 166 (36.6%), hepatic impairment in 12 (2.6%), interstitial lung disease in 6 (1.3%), and autoimmune disease in 4 (0.9%).

**TABLE 1:** Patient baseline characteristics at the start of avelumab maintenance therapy.

Variable	*N* = 453
Age, median (range), years	73 (21–91)
≤ 64 years, *n* (%)	75 (16.6)
65–74 years, *n* (%)	198 (43.7)
≥ 75 years, *n* (%)	180 (39.7)
BMI, median (range), kg/m^2^	22.8 (9.8–33.9)
Sex, *n* (%)	
Male	333 (73.5)
Female	120 (26.5)
ECOG performance status, *n* (%)	
0	325 (71.7)
1	111 (24.5)
≥ 2	17 (3.8)
Primary tumor site, *n* (%)	
Bladder	244 (53.9)
Upper urinary tract	209 (46.1)
Metastatic lesion, *n* (%)	
No	44 (9.7)
Yes	409 (90.3)
Site of metastasis, *n* (%)^a^	
Lung	109 (24.1)
Bone	55 (12.1)
Liver	39 (8.6)
Lymph node	309 (68.2)
Other	50 (11.0)
Disease stage, *n* (%)	
III	28 (6.2)
IV	404 (89.2)
Other	21 (4.6)
Comorbidities, *n* (%)^b^	
Renal impairment	166 (36.6)
Hepatic impairment	12 (2.6)
Interstitial lung disease	6 (1.3)
Autoimmune disease	4 (0.9)
Prior treatment, *n* (%)	
Surgery	333 (73.5)
Radiation therapy	63 (13.9)
Prior chemotherapy regimen, *n* (%)	
Gemcitabine + cisplatin	267 (58.9)
Gemcitabine + carboplatin	163 (36.0)
ddMVAC	9 (2.0)
Not reported or unknown	14 (3.1)
Number of cycles of prior chemotherapy, *n* (%)	
≤ 3	85 (18.8)
4	175 (38.6)
5	33 (7.3)
6	76 (16.8)
≥ 7	84 (18.5)
Best overall response to prior chemotherapy, *n* (%)	
CR	47 (10.4)
PR	242 (53.4)
SD	149 (32.9)
Not reported or unknown	15 (3.3)
Treatment‐free interval, *n* (%)	
< 4 weeks	204 (45.0)
4 to < 6 weeks	96 (21.2)
6 to < 8 weeks	67 (14.8)
8–10 weeks	26 (5.7)
> 10 weeks	60 (13.2)

Abbreviations: BMI, body mass index; CR, complete response; ddMVAC, dose‐dense methotrexate, vinblastine sulfate, doxorubicin hydrochloride, and cisplatin; ECOG, Eastern Cooperative Oncology Group; PR, partial response; SD, stable disease.

^a^ Patients with more than one metastatic site are included in more than one row.

^b^ Patients with more than one comorbidity were included.

**FIGURE 1:**
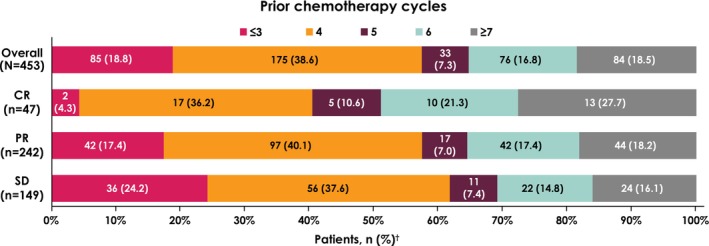
Number of prior chemotherapy cycles prior to starting avelumab maintenance therapy by best overall response to chemotherapy. The subgroup of patients whose best overall response was not reported or unknown (*n* = 15) is not shown. Abbreviations: CR, complete response; PR, partial response; SD, stable disease. ^†^Percentages may not total 100% due to rounding.

Prior chemotherapy regimen before avelumab maintenance therapy was gemcitabine + cisplatin in 267 patients (58.9%), gemcitabine + carboplatin in 163 (36.0%), and dose‐dense methotrexate, vinblastine, doxorubicin, and cisplatin in 9 (2.0%); in 14 patients (3.1%), prior chemotherapy was unknown (Table 1). Number of prior chemotherapy cycles was ≤ 3 cycles in 85 patients (18.8%), 4 cycles in 175 (38.6%), 5 cycles in 33 (7.3%), 6 cycles in 76 (16.8%), and ≥ 7 cycles in 84 (18.5%) (Figure 1). Best overall response to prior chemotherapy was complete response (CR) in 47 patients (10.4%), partial response (PR) in 242 (53.4%), and stable disease (SD) in 149 (32.9%). Number of prior treatment cycles in subgroups with CR, PR, or SD after prior chemotherapy is shown in Figure 1. The treatment‐free interval between the last dose of chemotherapy and first dose of avelumab was < 4 weeks in 204 patients (45.0%), 4–10 weeks in 189 (41.7%), and > 10 weeks in 60 (13.2%). Median duration of avelumab maintenance therapy was 5.1 months (interquartile range [IQR], 2.3–12.0 months), and median number of infusions was 10 (IQR, 4.0–21.0).

### 3.2 Safety

**FIGURE 2:**
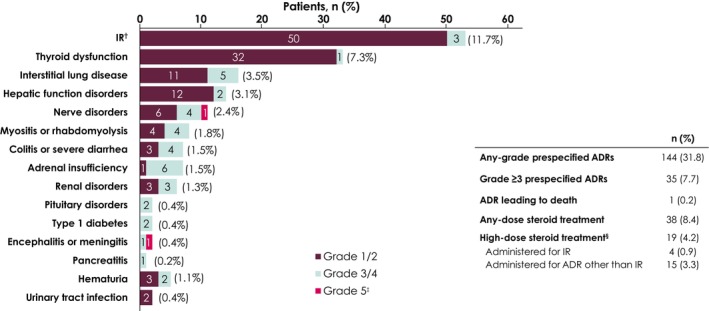
Prespecified ADRs (*N* = 453). Abbreviations: ADR, adverse drug reaction; IR, infusion reaction. ^†^IR includes infusion‐related reactions, chills, and pyrexia. ^‡^One grade 5 ADR was observed in one patient (noninfective encephalitis); encephalitis was categorized as a prespecified ADR for both nerve disorder and encephalitis in one patient and was counted twice. ^§^Patients received ≥ 40 mg total daily dose of prednisone or its equivalent.

**FIGURE 3:**
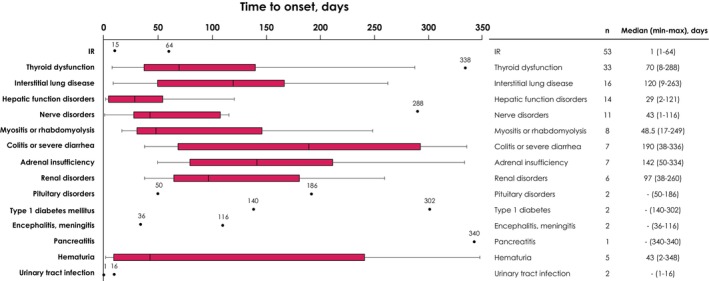
Time to onset of prespecified ADRs. Boxes represent IQR range (25th percentile to the 75th percentile), and vertical black lines within each box represent the median. Whiskers represent the lower and higher values within 1.5 × IQR, and dots represent outliers. Abbreviations: ADR, adverse drug reaction; IR, infusion reaction; max, maximum; min, minimum; IQR, interquartile range.

Among the 453 patients in the safety analysis population, prespecified ADRs were observed at any grade in 144 patients (31.8%) and grade ≥ 3 in 35 patients (7.7%). The most common any‐grade prespecified ADRs (≥ 5% of patients) were IRs in 53 patients (11.7%) and thyroid dysfunction in 33 patients (7.3%) (Figure 2). The most common grade 3/4 prespecified ADRs (≥ 1% of patients) were adrenal insufficiency in 6 patients (1.3%) and interstitial lung disease in 5 patients (1.1%). One grade 5 ADR was observed in 1 patient (noninfective encephalitis), which was categorized as a prespecified ADR for both nerve disorder and encephalitis. The day of onset of prespecified ADRs was day 1 in 53 patients (11.7%; IR in 51 patients), days 2–14 in 7 (1.5%), days 15–28 in 13 (2.9%), days 29–84 in 44 (9.7%), and day ≥ 85 in 52 (11.5%) (Figure S1). Median time to onset of specific prespecified ADRs is shown in Figure 3.

Of the 53 patients who had prespecified ADRs that were classified as an IR, initial occurrence (at any grade) was at first infusion in 51 patients (96.2%) and at second or fourth infusions in 1 patient (1.9%) each (Figure S2). Three patients (5.7%) had 2 IRs and 3 patients (5.7%) had grade 3 IRs at the first infusion. Timing of IR onset from start of infusion was immediately after the start of infusion in 4 patients (7.6%), during infusion in 16 (30.2%), and at ≤ 1 h or > 1 h after the end of infusion in 9 (17.0%) and 24 (45.3%), respectively. Premedication was administered to 447 patients (98.7%) at first dose, of whom 50 (11.2%) had an IR; of 6 patients who did not receive premedication, 1 (16.7%) had an IR (Table S1). Any‐dose corticosteroid treatment for ADRs was administered for prespecified ADRs in 38 patients (8.4%), of whom high‐dose corticosteroid treatment (≥ 40 mg total daily dose of prednisone or equivalent) was administered to 19 patients (4.2%), which was for an IR in 4 (0.9%) and prespecified ADRs other than IR in 15 (3.3%).

### 3.3 Effectiveness

**FIGURE 4:**
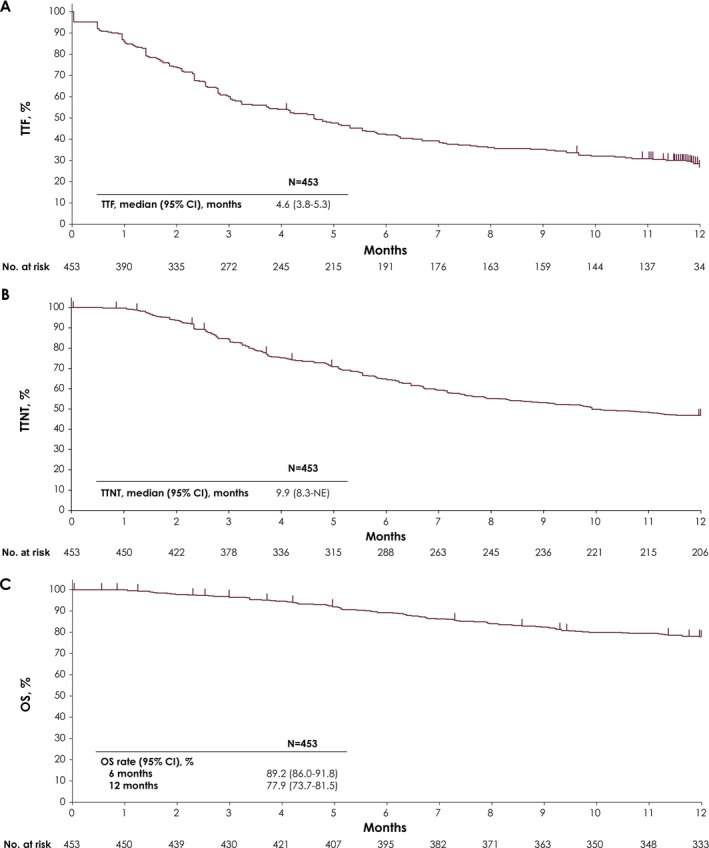
Kaplan–Meier analysis of (A) TTF, (B) TTNT, and (C) OS from the start of avelumab maintenance therapy (*N* = 453). Abbreviations: CI, confidence interval; NE, not estimable; OS, overall survival; TTF, time to treatment failure; TTNT, time to next‐line treatment.

Among the 453 patients in the effectiveness analysis population, median TTF from the start of avelumab maintenance therapy was 4.6 months (95% CI, 3.8–5.3 months) (Figure 4A). Median TTNT from the start of avelumab maintenance therapy was 9.9 months (95% CI, 8.3 months—not estimable) (Figure 4B). Median OS from the start of avelumab maintenance therapy was not reached; OS rates at 6 and 12 months were 89.2% (95% CI, 86.0%–91.8%) and 77.9% (95% CI, 73.7%–81.5%), respectively (Figure 4C).

### 3.4 Treatment Disposition and Next‐Line Treatment

At data cutoff, 131 patients (28.9%) were still receiving avelumab maintenance therapy and 322 patients (71.1%) had discontinued avelumab (Table 2). The most common reason for treatment discontinuation was disease progression in 225 patients (49.7%), followed by prespecified ADRs in 32 patients (7.1%). In total, 184 patients (57.1% of patients who discontinued avelumab) received next‐line anticancer drug treatment after avelumab maintenance therapy, including 154 of 225 patients (68.4%) who discontinued because of disease progression and 13 of 32 patients (40.6%) who discontinued because of prespecified ADRs (Table 3). The most common next‐line treatments (≥ 20% of patients [*n* = 184]) were gemcitabine + cisplatin in 53 patients (28.8%) and enfortumab vedotin in 44 (23.9%).

**TABLE 2:** Treatment disposition at data cutoff.

	*N* = 453
Avelumab maintenance therapy ongoing, *n* (%)	131 (28.9)
Avelumab maintenance therapy discontinued, *n* (%)	322 (71.1)
Reason for discontinuation, *n* (%)^a^	
Disease progression	225 (49.7)
Prespecified ADRs	32 (7.1)
Surgery	7 (1.5)
Patient preference	25 (5.5)
Death	4 (0.9)
Transfer to another hospital	5 (1.1)
Other	32 (7.1)

Abbreviation: ADR, adverse drug reaction.

^a^ Patients with more than one reason stated for discontinuation are included in more than one row.

**TABLE 3:** Subsequent treatment in the overall population and in subgroups of patients who discontinued because of progressive disease or prespecified ADRs.

	Patients who discontinued avelumab maintenance therapy (*n* = 322)^a^	Reason for discontinuation	
		Progressive disease (*n* = 225)	Prespecified ADRs (*n* = 32)
Received subsequent anticancer drug treatment, *n* (%)^c^	184 (57.1)^b^	154 (68.4)	13 (40.6)
Type of subsequent treatment, *n* (%)^d^			
Chemotherapy	109 (59.2)	89 (57.8)	8 (61.5)
Gemcitabine + cisplatin	53 (28.8)	43 (27.9)	3 (23.1)
Gemcitabine + carboplatin	34 (18.5)	29 (18.8)	3 (23.1)
Chemotherapy (other combination)	14 (7.6)	13 (8.4)	1 (7.7)
Chemotherapy (monotherapy)	8 (4.3)	4 (2.6)	1 (7.7)
Enfortumab vedotin	44 (23.9)	39 (25.3)	3 (23.1)
Pembrolizumab	30 (16.3)	25 (16.2)	2 (15.4)
Other	1 (0.5)	1 (0.6)	0

Abbreviation: ADR, adverse drug reaction.

^a^ Includes 68 patients who discontinued for reasons other than disease progression or prespecified ADRs.

^b^ Includes 17 patients who discontinued for reasons other than disease progression or prespecified ADRs.

^c^ Percentage calculated using the total number of patients who discontinued avelumab (overall or in the subgroups shown).

^d^ Percentage calculated using the total number of patients who received subsequent treatment (overall or in the subgroups shown).

## 4 Discussion

This analysis of PMS data provides a large and comprehensive study of patients with curatively unresectable or metastatic UC treated with avelumab maintenance therapy in real‐world clinical practice in Japan. Overall, the safety and effectiveness of avelumab in this PMS were comparable to findings from the JAVELIN Bladder 100 phase 3 trial and real‐world studies in various countries [16, 17, 18, 19, 20, 21, 22, 23, 24, 25]. ADRs were manageable, and the rates of ADRs and time to onset were consistent with expectations, confirming the acceptable tolerability of avelumab maintenance therapy.

The PMS patient population had differences in demographic and disease characteristics compared with the patient population in the avelumab + BSC arm of JAVELIN Bladder 100, including higher proportions of patients aged ≥ 65 years (83.4% vs. 63.1%) or ≥ 75 years (39.7% vs. 24.3%) and a higher proportion with a primary tumor site in the upper urinary tract (46.1% vs. 30.3%) [16, 31]. The PMS population also included patients who would not have been eligible for JAVELIN Bladder 100, including patients with an ECOG performance status of ≥ 2 (3.8%) or stage III disease (6.2%) [16]. Despite the more heterogeneous population, the safety profile of avelumab maintenance therapy in the PMS was comparable to that observed in JAVELIN Bladder 100 [16, 17, 18], and no additional safety concerns were identified. The most common any‐grade prespecified ADRs in the PMS population were IRs and thyroid dysfunction, which occurred in proportions comparable to those in JAVELIN Bladder 100 (IR in 11.7% vs. infusion‐related reaction in 21.5% and thyroid dysfunction in 7.3% vs. 16.6%, respectively) [16]. Prescribing information for avelumab recommends that patients should receive premedication with an antihistamine and antipyretic analgesics before receiving avelumab to mitigate the risk of IRs [28]; in the PMS population, 98.7% of patients received premedication, indicating strong adherence to this recommendation in Japan. The proportion of patients who received high‐dose corticosteroids was smaller in the PMS than in JAVELIN Bladder 100 (4.2% vs. 9.0%, respectively) [16]. Overall, these results further support the safety and tolerability of avelumab maintenance therapy in day‐to‐day clinical practice.

The Clinical Practice Guidelines for Bladder Cancer, developed under the instruction of the Japanese Urological Association, do not specify the number of cycles of platinum‐based chemotherapy that patients should receive, including guidelines published before or after the approval of avelumab maintenance therapy in Japan [29]. In the PMS population, 62.7% of patients had received 4–6 cycles of prior chemotherapy, and the most common number of cycles received was 4 (38.6%). Similarly, in subgroups defined by best overall response to prior chemotherapy, 36.2%–40.1% of patients who had a CR, PR, or SD had received 4 cycles. In the overall PMS population, 18.8% of patients had received ≤ 3 cycles of prior chemotherapy and 18.5% had received ≥ 7 cycles; the subgroup who received ≥ 7 cycles may have included some patients who started chemotherapy prior to the approval of avelumab then switched to avelumab after approval. In clinical practice, treating physicians may switch to avelumab maintenance therapy as appropriate based on individual patient and disease characteristics. A separate manuscript reports detailed analyses from the PMS population in subgroups defined by age, prior chemotherapy regimen, and best overall response to prior chemotherapy [32].

In the PMS population, the effectiveness of avelumab maintenance therapy was observed in routine clinical practice in Japan. Median TTF in this population (4.6 months) was similar to that reported in a smaller real‐world study performed in Japan (4.6 months; *N* = 79) [21]. In addition, the 12‐month OS rate (77.9%) was comparable to rates in the JAVELIN Bladder 100 trial (71.3%) and real‐world studies in other countries (63.0%–75.7%) [16, 22, 23, 24, 25, 26]. Patient characteristics varied between the PMS population and those in other real‐world studies (where reported), such as for median age (PMS, 73 years; other studies, 69–73 years), proportions with upper tract primary tumors (46.1% vs. 14.8%–30.6%), proportions that received prior carboplatin + gemcitabine (36.0% vs. 34.3%–61.5%), or proportions with a best response to chemotherapy of CR or PR (63.8% vs. 66.9%–85.6%) [22, 23, 24, 25, 26]. Comparisons between different real‐world studies should be interpreted with caution because of differences in study designs, healthcare systems in each country, and patient backgrounds. However, acknowledging these differences, our findings suggest that avelumab maintenance therapy is an effective treatment for heterogeneous real‐world populations of patients with UC without disease progression after platinum‐based chemotherapy, including patients with differences in demographic and disease characteristics compared with the JAVELIN Bladder 100 trial population.

To our knowledge, this PMS provides the first report of TTNT from initiation of avelumab maintenance therapy in Japan (median, 9.9 months). Recently, other studies have reported TTNT in patients with advanced UC. In a retrospective study in the US using data from electronic health records of patients who received avelumab maintenance therapy, median TTNT from initiation of avelumab was 7.0 months [25]. Additionally, in a real‐world study using electronic medical records of patients with advanced UC who received various 1L options in Japan, median TTNT from start of 1L therapy was 9.0 months [33]. TTNT has been proposed as a surrogate endpoint for OS in patients with advanced melanoma, renal cell carcinoma, and non‐small cell lung cancer treated with immune checkpoint inhibitors (ICIs) [34, 35, 36]. TTNT includes the treatment‐free period (i.e., the period of time between the end of a treatment and the start of subsequent treatment), allowing for a more comprehensive evaluation of benefit from ICI treatment [34, 35, 36, 37]. Because ICIs alter the immune system, clinical benefit may continue after ICI discontinuation, and a prolonged treatment‐free period may be a positive indicator of overall disease stability; this may be a consideration in the decision to initiate subsequent treatment [37, 38, 39, 40]. However, further studies are needed to evaluate the relevance of TTNT, including the treatment‐free period, as indicators of clinical benefit in patients with advanced UC.

The potential to receive next‐line therapy may depend on reasons for discontinuation. In this study, the rate of transition to next‐line subsequent treatment was lower in patients who discontinued because of prespecified ADRs (40.6%) than in those who discontinued because of progressive disease (68.4%), suggesting that the proper management of ADRs is important for next‐line therapy. However, irrespective of whether patients discontinued avelumab because of prespecified ADRs or disease progression, next‐line treatment was commonly received and most frequently involved platinum‐based chemotherapy or enfortumab vedotin monotherapy. It should be noted that enfortumab vedotin did not receive regulatory approval until after the PMS had been initiated [41]; thus, available post‐avelumab treatment options were not identical for all patients in the PMS population.

While this PMS was performed in accordance with GPSP, the data reported here have several limitations. Data were collected from CRFs and were not reviewed by an independent data monitoring committee. Methods of patient assessment in the PMS were different from those used in clinical trials. Additionally, the PMS was a noninterventional, observational study with no control group and had a limited observation period of 52 weeks; therefore, it was not possible to confirm that patient outcomes observed were due to avelumab treatment. Comparisons with JAVELIN Bladder 100 data must be interpreted with caution.

## 5 Conclusions

Overall, the safety, tolerability, and effectiveness of avelumab maintenance therapy observed in this large PMS population in Japan was generally consistent with reports from previous clinical studies, despite the heterogeneity of patient characteristics. Findings demonstrate the favorable benefit–risk profile of avelumab maintenance therapy in patients with advanced UC and support its continued use in clinical practice.

## Author Contributions

**Eiji Kikuchi:** conceptualization, visualization, supervision, writing – review and editing. **Masayoshi Nagata:** conceptualization, supervision, writing – review and editing. **Taito Ito:** investigation, methodology, formal analysis, writing – review and editing. **Masashi Sato:** investigation, methodology, software, formal analysis, writing – review and editing. **Mie Ogi:** investigation, methodology, data curation, writing – review and editing. **Makiko Morita:** investigation, methodology, validation, writing – review and editing. **Masahiro Kajita:** conceptualization, visualization, writing – original draft preparation, writing – review and Editing. **Hiroyuki Nishiyama:** conceptualization, supervision, writing – review and editing.

## Ethics Statement

This study was conducted in accordance with the regulations of the GPSP Ministerial Ordinance in Japan. Approval from ethics committees/institutional review boards and informed consent for publication from patients were obtained based on the requirements of each institution.

## Conflicts of Interest

Eiji Kikuchi reports providing consulting or advisory roles for Astellas Pharma, AstraZeneca, Bristol Myers Squibb‐Ono Pharmaceutical, Chugai Pharmaceutical, Janssen, the healthcare business of Merck KGaA, Darmstadt, Germany, Merck & Co., Kenilworth, NJ, and Pfizer; speakers bureau participation for Astellas Pharma, AstraZeneca, Bayer, Bristol Myers Squibb‐Ono Pharmaceutical, Chugai Pharmaceutical, Janssen, Kissei Pharmaceutical, Kyorin, Kyowa Kirin International, the healthcare business of Merck KGaA, Darmstadt, Germany, Merck & Co., Kenilworth, NJ, Nippon Kayaku, Nippon Shinyaku, Pfizer, Sanofi, Taiho Pharmaceutical, and Takeda; and institutional research funding from Chugai Pharmaceutical, Kissei Pharmaceutical, Kyorin, Kyowa Kirin International, Nippon Kayaku, Nippon Shinyaku, Otsuka, Sanofi, Taiho Pharmaceutical, and Takeda. Masayoshi Nagata reports speakers bureau participation for Janssen and Sanofi. Taito Ito, Masashi Sato, Mie Ogi, Makiko Morita, and Masahiro Kajita report employment with Merck Biopharma Co., Ltd., Tokyo, Japan, an affiliate of Merck KGaA, Darmstadt, Germany. Hiroyuki Nishiyama reports speakers bureau participation for Astellas Pharma, Bristol Myers Squibb, the healthcare business of Merck KGaA, Darmstadt, Germany, Merck & Co., Kenilworth, NJ, and Ono Pharmaceutical; and research funding from Astellas Pharma and Chugai Pharmaceutical.

## Supporting information


**Data S1:** cam471264‐sup‐0001‐DataS1.docx.

## Data Availability

Any requests for data by qualified scientific and medical researchers for legitimate research purposes will be subject to the healthcare business of Merck KGaA, Darmstadt, Germany's Data Sharing Policy. All requests should be submitted in writing to the healthcare business of Merck KGaA, Darmstadt, Germany's data sharing portal (https://www.emdgroup.com/en/research/our‐approach‐to‐research‐and‐development/healthcare/clinical‐trials/commitment‐responsible‐data‐sharing.html). When the healthcare business of Merck KGaA, Darmstadt, Germany has a co‐research, co‐development, or co‐marketing or co‐promotion agreement, or when the product has been out‐licensed, the responsibility for disclosure might be dependent on the agreement between parties. Under these circumstances, the healthcare business of Merck KGaA, Darmstadt, Germany will endeavor to gain agreement to share data in response to requests.
