# Supplementary material for: Real‐World Outcomes of Avelumab Maintenance Therapy in Patients With Curatively Unresectable Urothelial Carcinoma in Japan: Results From the Final Analysis of Postmarketing Surveillance
Source: Cancer Med. 2025 Oct 30;14(21):e71264. doi: 10.1002/cam4.71264 (PMC12572945; doi:10.1002/cam4.71264)

**SUPPORTING INFORMATION**

**Table S1.** Use of premedication prior to the first dose of avelumab (N=453) and occurrence of infusion reaction by type of premedication.

|  | **n (%)** | **Proportion with IR, n/N (%)** |
| --- | --- | --- |
| Premedication administered | 447 (98.7) | 50/447 (11.2) |
| Acetaminophen and diphenhydramine | 295 (65.1) | 29/295 (9.8) |
| Acetaminophen and chlorpheniramine maleate | 386 (19.0) | 15/386 (3.9) |
| Other | 66 (14.6) | 6/66 (9.1) |
| No premedication administered | 6 (1.3) | 1/6 (16.7) |

**IR,** infusion reaction.

**Figure S1.** Onset of prespecified ADRs by day.

**ADR**, adverse drug reaction; **IR**, infusion reaction**.**

**
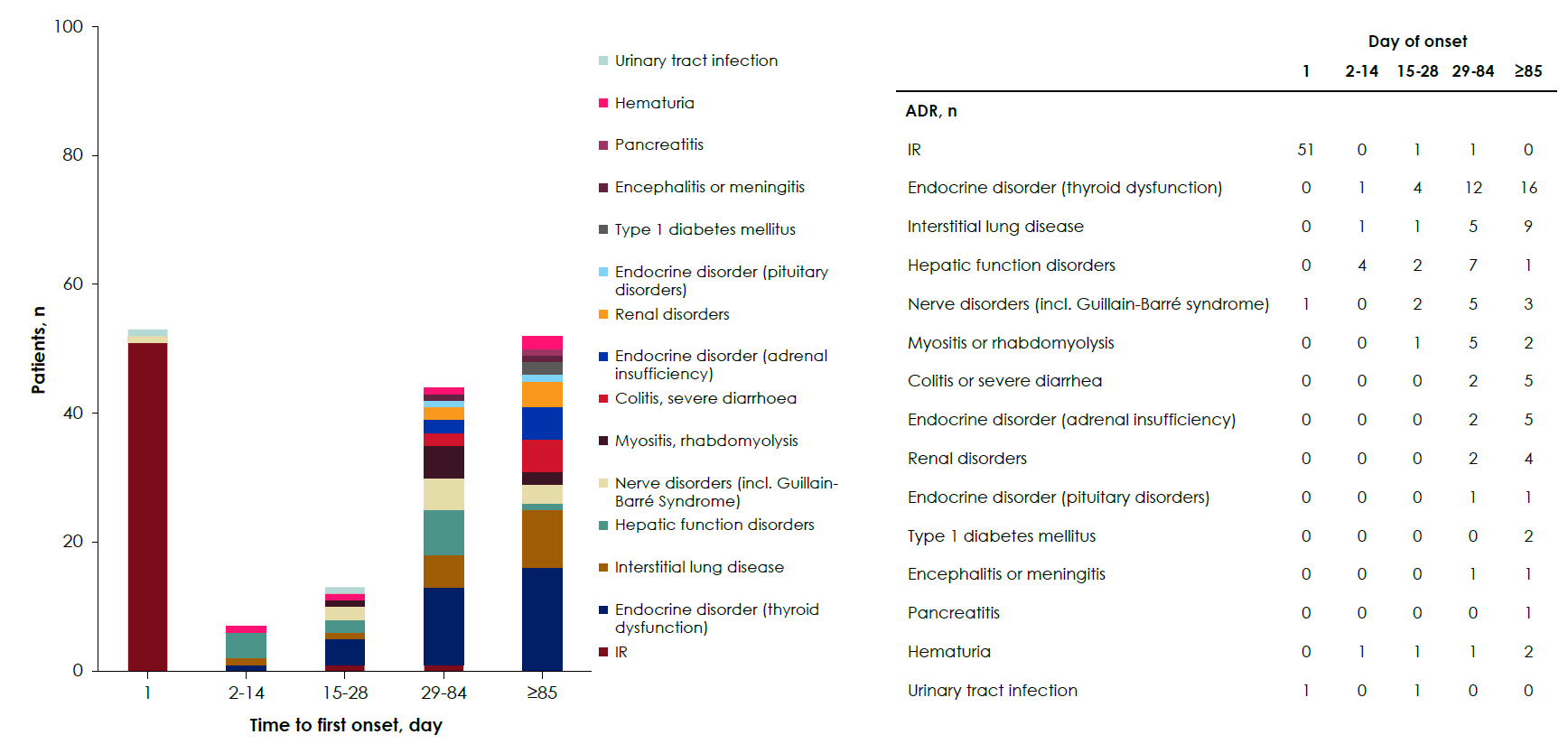
**

**Figure S2.** Time to onset of first IR by infusion number (n=53) and timing of IR onset from start of infusion.

**IR**, infusion reaction.


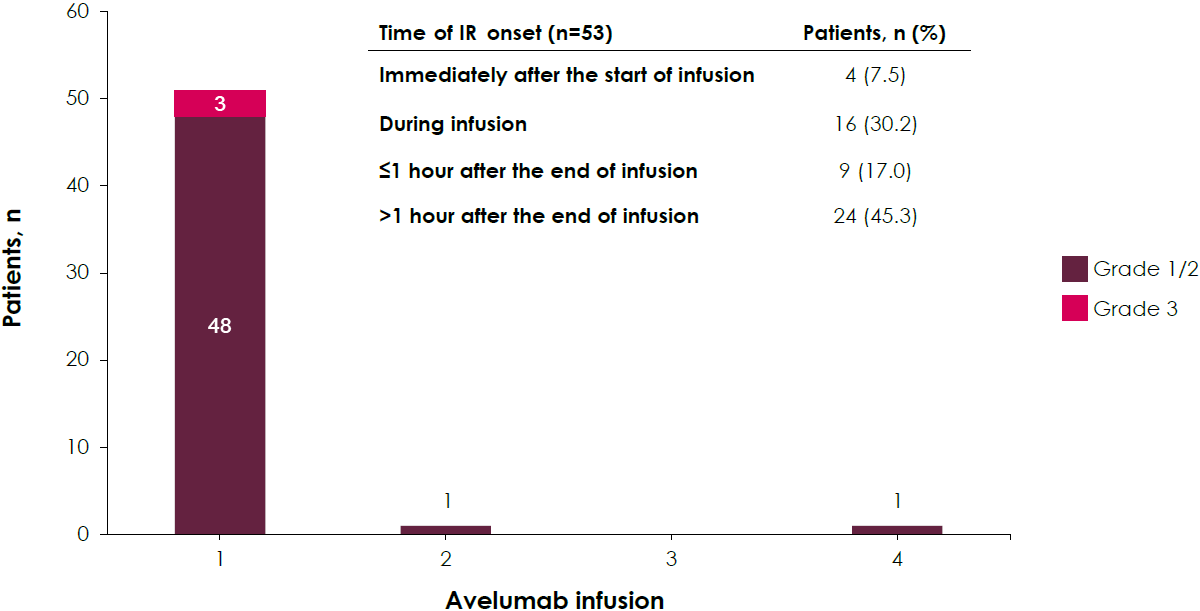

Supplement: Supplementary file 1 — Data S1: cam471264‐sup‐0001‐DataS1.docx. [file CAM4-14-e71264-s001.docx]
